# Transient YAP activation uncovers the neurogenic potential of proliferative mammalian Müller glia

**DOI:** 10.1093/pnasnexus/pgag188

**Published:** 2026-05-28

**Authors:** English J Laserna, Irina V Saltykova, Benjamin M Hall, Xuefei Tong, Justin S Dhindsa, Borna Sarker, Ayrea E Hurley, Paul G Swinton, William R Lagor, Nicholas M Tran, James F Martin, Ross A Poché

**Affiliations:** Genetics and Genomics Graduate Program, Baylor College of Medicine, Houston, TX 77030, USA; Department of Integrative Physiology, Baylor College of Medicine, Houston, TX 77030, USA; Department of Integrative Physiology, Baylor College of Medicine, Houston, TX 77030, USA; Department of Integrative Physiology, Baylor College of Medicine, Houston, TX 77030, USA; Department of Integrative Physiology, Baylor College of Medicine, Houston, TX 77030, USA; Genetics and Genomics Graduate Program, Baylor College of Medicine, Houston, TX 77030, USA; Department of Human and Molecular Genetics, Baylor College of Medicine, Houston, TX 77030, USA; Department of Human and Molecular Genetics, Baylor College of Medicine, Houston, TX 77030, USA; Department of Integrative Physiology, Baylor College of Medicine, Houston, TX 77030, USA; Department of Integrative Physiology, Baylor College of Medicine, Houston, TX 77030, USA; The Texas Heart Institute, Baylor College of Medicine, Houston, TX 77030, USA; Genetics and Genomics Graduate Program, Baylor College of Medicine, Houston, TX 77030, USA; Department of Integrative Physiology, Baylor College of Medicine, Houston, TX 77030, USA; Genetics and Genomics Graduate Program, Baylor College of Medicine, Houston, TX 77030, USA; Department of Human and Molecular Genetics, Baylor College of Medicine, Houston, TX 77030, USA; Genetics and Genomics Graduate Program, Baylor College of Medicine, Houston, TX 77030, USA; Department of Integrative Physiology, Baylor College of Medicine, Houston, TX 77030, USA; The Texas Heart Institute, Baylor College of Medicine, Houston, TX 77030, USA; Center for Organ Repair and Renewal, Baylor College of Medicine, Houston, TX 77003, USA; Genetics and Genomics Graduate Program, Baylor College of Medicine, Houston, TX 77030, USA; Department of Integrative Physiology, Baylor College of Medicine, Houston, TX 77030, USA; Center for Organ Repair and Renewal, Baylor College of Medicine, Houston, TX 77003, USA

**Keywords:** regeneration, mammalian retina, Müller glia, Hippo signaling, AAV

## Abstract

The Hippo pathway effector YAP promotes spontaneous proliferation of Müller glia (MG), suggesting that bypassing Hippo signaling and activating YAP could enhance retinal regeneration. However, whether proliferative adult MGs retain meaningful neurogenic competence remains unclear. Here, using viral delivery of a Hippo-resistant YAP variant to wild-type adult MGs, we achieved transient YAP activation in adult MGs, inducing proliferation followed by cell-cycle withdrawal and differentiation. Intersectional genetic lineage tracing and EdU labeling, combined with transcriptomic analyses, revealed that YAP-activated MGs predominantly regenerate MGs, whereas only a subset gives rise to bipolar cell-like neurons. These results indicate that proliferative MGs acquire a state resembling that of late-stage retinal progenitors, with limited neurogenic lineage potential. We conclude that YAP-activated cell-cycle reentry inefficiently reprograms adult MGs toward photoreceptor or ganglion cell fates. These findings define the limited competence of proliferative adult MGs to contribute to neurogenic fates and provide a rigorous framework for assessing in vivo glial reprogramming strategies.

Significance Statement Adult mammalian Müller glia (MG) can be induced to proliferate; however, whether proliferation restores neurogenic competence remains uncertain. By transiently bypassing Hippo-mediated repression of YAP and using rigorous intersectional lineage tracing, we demonstrate that proliferative adult MGs predominantly regenerate MGs, whereas bipolar cell-like neurons are generated infrequently. These results indicate that YAP-mediated cell-cycle reentry primarily promotes MG self-renewal and secondarily induces bipolar-like cells, suggesting that further optimization could reestablish retinal progenitor potential. Our findings define a limited but measurable neurogenic competence in proliferative MGs and provide a framework for evaluating in vivo glial reprogramming strategies for retinal regeneration.

## Introduction

In zebrafish, Müller glial cells (MGs) reprogram into retinal progenitor cells that regenerate functional neurons following injury (1–6). In contrast, mammalian MGs exhibit limited regenerative capacity. It is thought that a major barrier to retinal regeneration in mammals is the failure of MGs to sustain proliferation. Whether proliferation alone is sufficient to unlock latent neurogenic competence in adult mammalian MGs remains a fundamental and unresolved question in regenerative biology.

We previously demonstrated that retinal injury activates the Hippo pathway effector YAP in mouse MGs, promoting cell-cycle entry via direct target genes, including *Cyclin D1* (7). However, Hippo-mediated phosphorylation rapidly inactivates YAP, resulting in MG cell-cycle withdrawal. Transgenic expression of a Hippo-nonresponsive YAP variant (YAP5SA) induces spontaneous MG proliferation and acquisition of a progenitor-like transcriptional state (7). However, constitutive YAP5SA expression maintains MGs in a proliferative state, precluding assessment of postmitotic differentiation and fate potential. Thus, it remains unclear whether proliferative adult MGs retain the capacity to generate neurons and whether their competence resembles embryonic retinal progenitors or is intrinsically fate-restricted. Definitive resolution of this question requires rigorous lineage tracing to establish MG-derived cell fates in vivo.

Here, we employ an adeno-associated virus (AAV)-based, intersectional genetic fate-mapping strategy to transiently activate YAP5SA in adult MGs and determine the identity of their daughter cells. We show that transient YAP activation induces proliferation followed by cell-cycle exit. Transcriptomic profiling reveals that proliferative MGs predominantly regenerate MGs, while flow cytometry analyses indicate limited bipolar cell neurogenic potential. These findings indicate that proliferative MGs have limited ability to reprogram to neurogenic fates and have implications for understanding how adult glia balance proliferation and lineage restriction across regenerative contexts.

## Results and discussion

### Lineage tracing of YAP5SA+ MG daughter cells

In our previous study, a CAG-driven *Yap5SA* transgene was activated in MG by tamoxifen-induced deletion of a floxed stop cassette using *Glast-CreERT2* (7). Constitutive *Yap5SA* expression held MGs in a proliferative state, preventing assessment of postmitotic daughter cell fate. To overcome this limitation, we developed a strategy to transiently activate *Yap5SA* in adult MGs, thereby inducing proliferation and enabling unambiguous tracing of the fate of postmitotic progeny.

We used AAV-mediated gene delivery to achieve transient *Yap5SA* expression. Because most AAV genomes remain episomal (∼0.1% integration frequency) (8–11), *Yap5SA* expression is expected to diminish as infected MGs divide and dilute episomes. For viral transduction, we selected the ShH10 capsid, a directed-evolution AAV6 variant reported to efficiently target MGs following intravitreal injection (12, 13). To assess tropism, we first delivered a *CBh-GFP* construct using ShH10 and observed GFP expression in multiple retinal cell types, including rods (Fig. S1A and B), indicating that capsid tropism alone was insufficient for MG specificity. Consistent with other studies, replacing the *CBh* promoter with a *Gfap* promoter markedly restricted GFP expression to MGs (Fig. S1C and D), and this configuration was used for subsequent experiments (14, 15).

**Figure 1:**
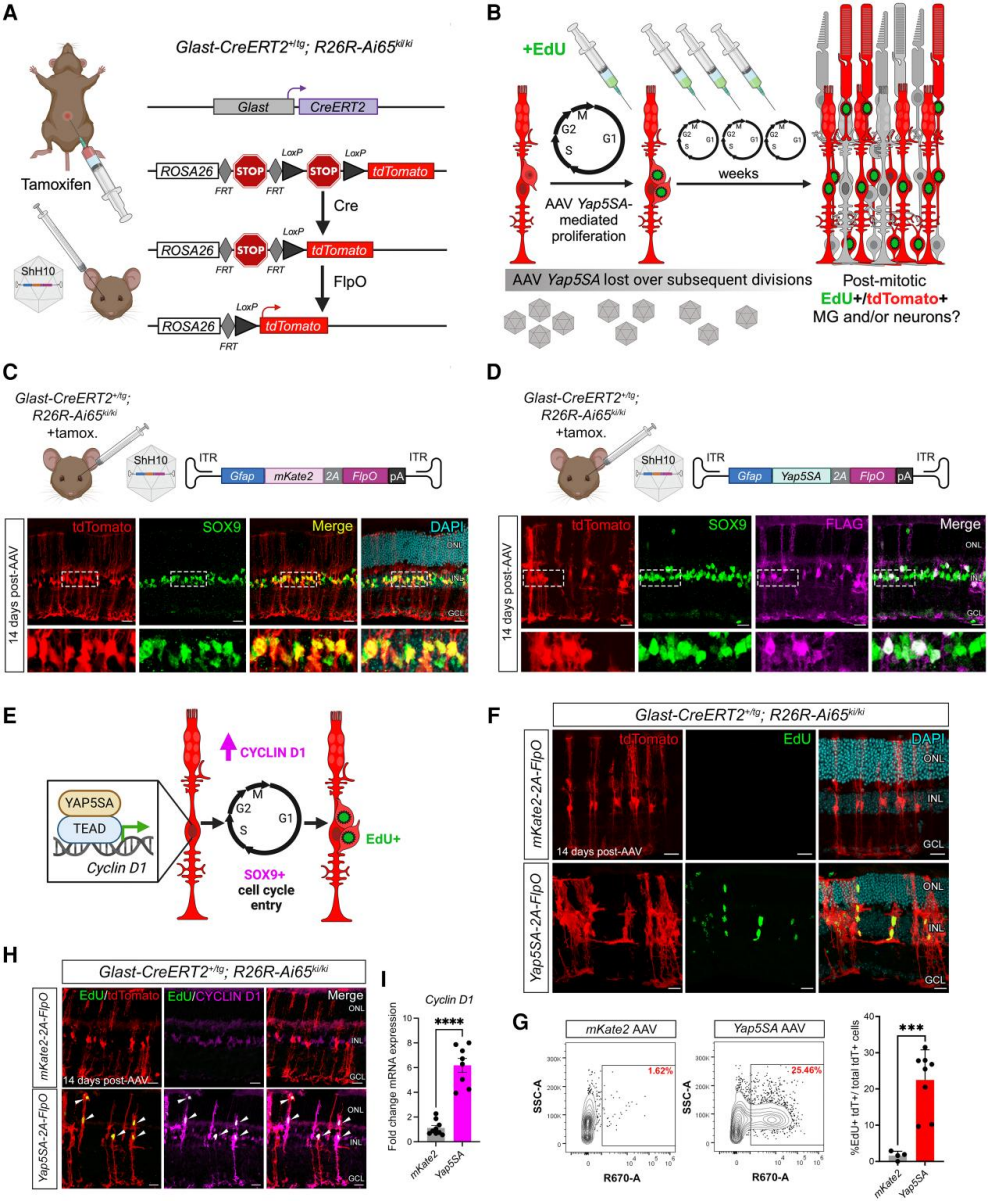
Intersectional genetic lineage tracing of proliferative YAP5SA+ MG cells. A and B) Schematic illustrating the overall experimental design. A) Adult *Glast-CreERT2^+/tg^; R26R-Ai65^ki/ki^* mice are injected with tamoxifen to induce Cre recombination of the loxP-flanked STOP cassette in the *R26R-Ai65* locus. Intravitreal delivery of AAV, encoding FlpO, recombines the FRT-flanked stop cassette, leading to tdTomato expression specifically in MG that received the AAV. B) If the AAV also carries *Yap5SA*, the tdTomato+ MGs will proliferate and incorporate EdU in their nuclei. Upon subsequent divisions, the cells will lose AAV particles, exit the cell cycle, but retain both the tdTomato and EdU labels as lineage markers for postmitotic daughter cells. C) Map of the *mKate2* negative control AAV and immunofluorescent (IF) image showing that, after 14 days postintravitreal injection, tdTomato is expressed in SOX9+ MGs. D) Map of the *Yap5SA* AAV and IF image showing that tdTomato and FLAG (fused to the N-terminus of YAP5SA) are expressed in SOX9+ MGs. E) Schematic summarizing how the previously published *Yap5SA* transgenic mouse drives MG spontaneous proliferation. F) EdU labeling showing that *Yap5SA* AAV induces spontaneous proliferation of tdTomato+ MGs, whereas *mKate2* AAV does not. G) Representative cytometry scatter plots and quantification showing a significant increase of tdTomato+/EdU+ cells in retinas infected with *Yap5SA* AAV. H) IF and I) qRT-PCR showing *Yap5SA* AAV-dependent upregulation of *Cyclin D1*. All quantified data are shown as mean ± SD and *n* ≥ 3 biologically independent samples per group. Significant differences between groups were determined using an unpaired t test with Welch correction. A *P*-value of <0.05 was considered significant. *P < 0.05, **P < 0.01, ***P < 0.001, ****P < 0.0001. Scale bars = 20 μm. Created in BioRender. Poché (2026) https://BioRender.com//9qf4bbm.

Adult *Glast-CreERT2^+/tg^*; *R26R-Ai65^ki/ki^* mice were intravitreally injected with ShH10 encoding either *Gfap-Yap5SA-2A-FlpO* or *Gfap-mKate2–2A-FlpO* (negative control). Following tamoxifen administration, Cre- and FlpO-mediated recombination activated tdTomato expression exclusively in infected MGs and their progeny (Fig. 1A). To independently track proliferating MGs, EdU was administered every other day until harvest. Because AAV episomes are diluted with cell division, postmitotic EdU+/tdTomato+ cells necessarily arise from *Yap5SA*-expressing MGs (Fig. 1B).

Fourteen days postinjection, tdTomato labeling in control retinas was confined to SOX9+ MGs (Fig. 1C). A similar pattern was observed following *Yap5SA* AAV infection (Fig. 1D). FLAG-tagged YAP5SA colocalized with tdTomato+/SOX9+ cells (Fig. 1D, inset), confirming expression in MGs. Immunofluorescence for IBA1, GFAP, and S100β did not overlap with tdTomato or FLAG (Fig. S1E, arrowheads), indicating no detectable infection of microglia or astrocytes. Together, these results demonstrate that ShH10, when combined with the *Gfap* promoter, provides substantial MG specificity and enables faithful lineage tracing of infected MGs and their progeny.

### *Yap5SA* AAV drives robust MG proliferation

We next asked whether AAV-mediated *Yap5SA* delivery induces proliferation comparable to that observed in the previously characterized *Yap5SA* transgenic model (7). Transgenic *Yap5SA* expression robustly upregulates *Cyclin D1* and drives persistent MG proliferation independent of injury (Fig. 1E) (7). Adult *Glast-CreERT2^+/tg^; R26R-Ai65^ki/ki^* retinas were infected with *Yap5SA* or control AAV and administered EdU every other day for 14 days. EdU incorporation was detected exclusively in *Yap5SA*-infected retinas and with tdTomato+ cells (Fig. 1F and G). Consistent with prior findings, *Yap5SA* expression markedly increased *Cyclin D1* levels (Fig. 1H and I) and occurred predominantly within SOX9+ MGs (Fig. S1F). Importantly, activated CASPASE-3 immunostaining was minimal in tdTomato+/EdU+ cells at 14, 56, and 112 days postinfection (Fig. S1G), indicating that proliferating MGs did not undergo apoptosis. Annexin V flow cytometry of infected wild-type retinas also failed to show a significant increase in apoptotic cells following *Yap5SA* AAV delivery (Fig. S1H and I). Together, these findings demonstrate that AAV-delivered *Yap5SA* robustly induces spontaneous MG proliferation without widespread cell death and recapitulates the proliferative phenotype observed in the previously reported transgenic model.

### *Yap5SA* AAV-driven MG proliferation is transient and yields postmitotic progeny

A key premise of this approach is that AAV genomes persist primarily as episomes and are not efficiently inherited during cell division. We therefore reasoned that *Yap5SA* expression would diminish as proliferating MGs dilute episomes, permitting cell-cycle exit, and enabling assessment of postmitotic fate.

**Figure 2:**
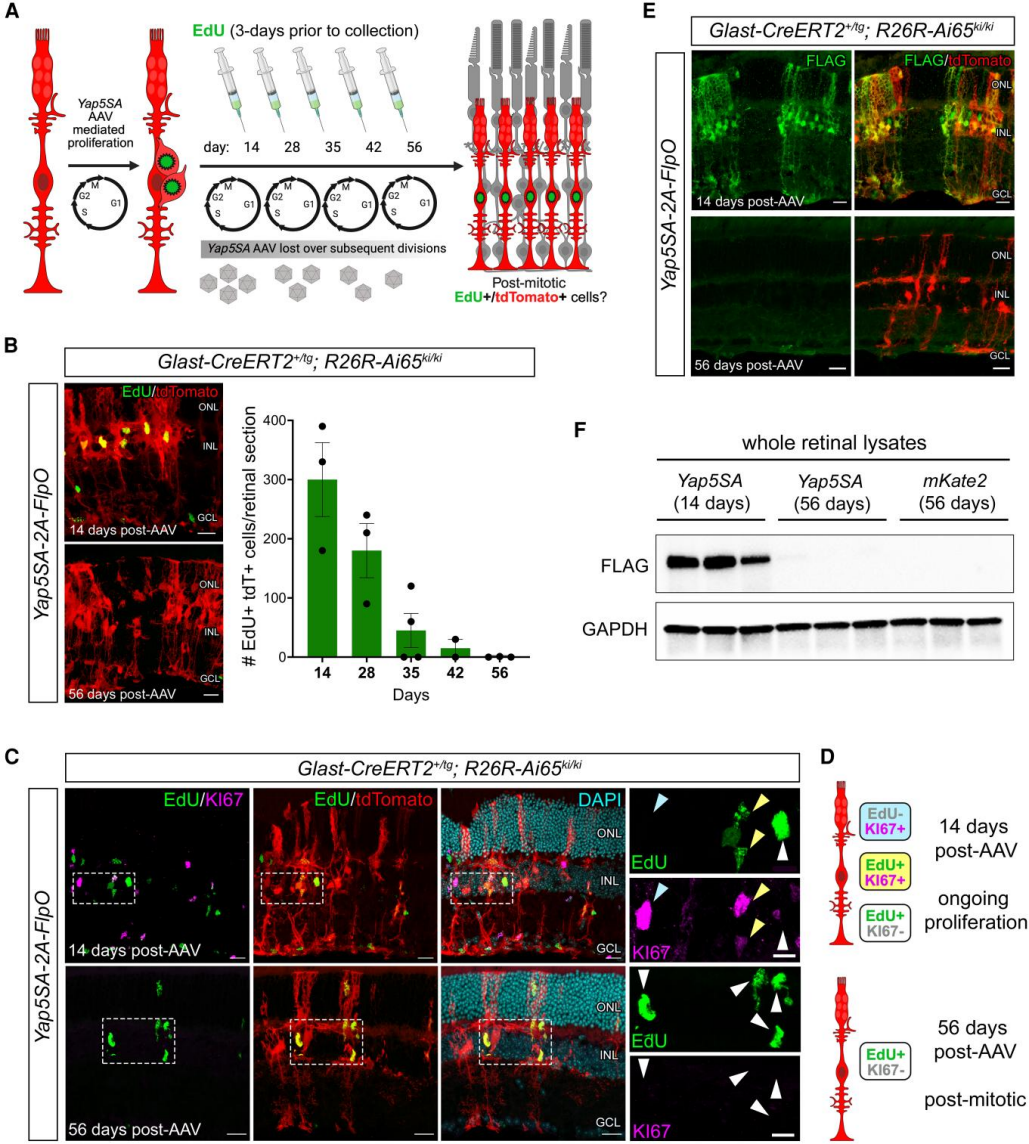
*Yap5SA* AAV-driven MG proliferation is transient. A) Schematic illustrating experimental design. B) Left: EdU labeling showing *Yap5SA* AAV-dependent MG proliferation 14 days postinfection and no labeling at 56 days postinfection. Right: Quantification of EdU+/tdTomato+ cells over time. C) Top row: IF labeling showing the presence of KI67+/EdU+/tdTomato+ cells actively in the cell cycle 14 days post-*Yap5SA* AAV (yellow arrowheads), likely a KI67+/tdTomato+ cell that has yet to take up EdU (blue arrowhead), and EdU+/tdTomato+ cells that are not labeled with KI67, suggesting cell-cycle exit (white arrowheads). Bottom row: IF labeling showing that by 56 days postinfection, KI67 labeling is absent from the EdU+/tdTomato+ cells, suggesting that most cells are now postmitotic (white arrowheads). For the retinas shown in (C), EdU was administered daily until the tissue was processed for histology. D) Schematic summary of results. E) IF showing FLAG-tagged YAP5SA in tdTomato+ MGs 14 days post-AAV and its absence at 56 days post-AAV. F) Western blot confirmation that YAP5SA::FLAG is lost by 56 days post-AAV infection. Scale bars = 20 μm. Created in BioRender. Poché (2026).

To test whether *Yap5SA* AAV induces transient proliferation, we performed a time-course analysis. Adult *Glast-CreERT2^+/tg^*; *R26R-Ai65^ki/ki^* mice were intravitreally injected with *Yap5SA* AAV and administered EdU for three consecutive days prior to harvest at 14, 28, 35, 42, and 56 days postinfection (Fig. 2A). At 14 days, EdU+/tdTomato+ cells were readily detected throughout the retina (Fig. 2B). In contrast, by 56 days, EdU incorporation was no longer observed, indicating cessation of MG proliferation (Fig. 2B). To distinguish cell-cycle exit from cell loss, upon AAV infection, EdU was administered daily for 14 or 56 days, and retinas were costained for EdU and KI67. At 14 days, numerous EdU+/KI67+/tdTomato+ cells were present, consistent with ongoing proliferation (Fig. 2C and D). By 56 days, tdTomato+ cells retained EdU labeling but no longer expressed KI67, demonstrating that these cells had exited the cell cycle and persisted as postmitotic progeny. Consistent with this interpretation, immunostaining and Western blot analysis revealed loss of FLAG-tagged YAP5SA protein by 56 days (Fig. 2E and F), coinciding temporally with the cessation of proliferation.

One possible explanation for the eventual loss of detectable YAP5SA expression is dilution of nonintegrating AAV genomes during MG proliferation. However, we did not determine the initial intracellular viral copy number, the number of MG divisions following transduction, or the residual vector copy number at later time points. We therefore cannot conclude that viral dilution is the sole mechanism underlying loss of expression. Alternative explanations, including transgene silencing, reduced promoter activity, or other forms of posttranscriptional downregulation, may also contribute. Together, these data demonstrate that AAV-delivered *Yap5SA* drives robust yet self-limited MG proliferation, and that loss of detectable YAP5SA expression coincides with cell-cycle exit and stabilization of postmitotic daughter cells, thereby providing an experimental window to assess their differentiation potential.

### MG daughter cells are predominantly lineage-restricted, with rare bipolar cell-like progeny

**Figure 3:**
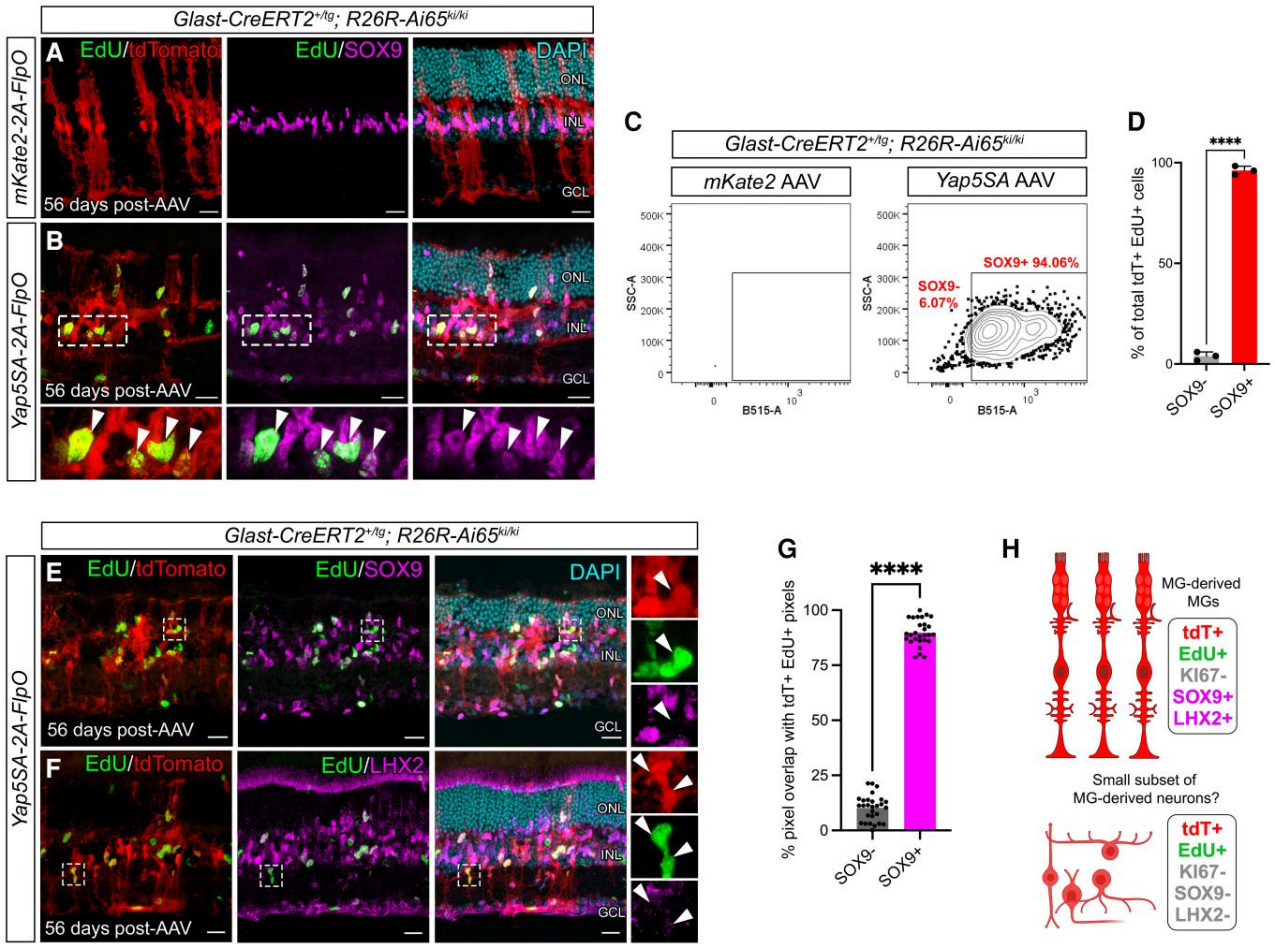
The postmitotic daughter cells of proliferative MG mostly express MG markers. A and B) IF for SOX9, identifying the majority of postmitotic EdU+/tdTomato+ cells at 56 days post-*Yap5SA* AAV as MGs. C and D) Representative cytometry scatter plots and quantification showing that most of the EdU+/tdTomato+ cells express SOX9, but a small percentage do not. E and F) IF confirmation of the presence of a discrete population of EdU+/tdTomato+ cells that do not express the MG markers SOX9 or LHX2 (boxed region and arrowheads). G) Quantification of the % overlap of EdU+/tdTomato+ pixels with SOX9+ or SOX9− pixels. H) Schematic summary of the results in (E–G). All quantified data are shown as mean ± SD and *n* ≥ 3 biologically independent samples per group. Significant differences between groups were determined using paired t tests. A *P*-value of <0.05 was considered significant. Scale bars = 20 μm. *P < 0.05, **P < 0.01, ***P < 0.001, ****P < 0.0001.Created in BioRender. Poché (2026) https://BioRender.com/k67x69s.

To determine the fate of postmitotic YAP5SA+ MG daughter cells, adult *CreERT2^+/tg^; R26R-Ai65^ki/ki^* mice were intravitreally injected with either control *(mKate2)* or *Yap5SA* AAV and administered EdU every other day for 56 days prior to analysis. As expected, control retinas showed no tdTomato+/SOX9+ MGs incorporating EdU. In contrast, *Yap5SA* AAV-infected retinas contained persistent tdTomato+/EdU+ cells that coexpressed SOX9 and LHX2, consistent with postmitotic MG identity (Figs. 3A and B and S2A and B). Flow cytometric quantification revealed that 96.13% ± 1.92 (mean ± SD) of the tdTomato+/EdU+ population retained SOX9 expression, whereas 3.86% ± 1.91 were SOX9-negative (Fig. 3C and D). Pixel-based image analysis yielded similar results, with 89.75% of EdU+/tdTomato+ signal co-localizing with SOX9 (Fig. 3G). These data indicate that the overwhelming majority of YAP5SA-induced MG daughter cells maintain MG identity. Nevertheless, a small but reproducible subset of tdTomato+/EdU+ cells lacked both SOX9 and LHX2 (Fig. 3E–G), suggesting that proliferative MGs may also generate nonglial progeny (Fig. 3H).

Previous studies have demonstrated direct transdifferentiation of adult MGs into inner retinal neurons, typically through forced expression of proneural transcription factors such as *Ascl1*, often combined with chromatin modulation and injury, and in the absence of preceding cell division (16–24). In contrast, our approach activates endogenous proliferative signaling by transiently expressing YAP5SA in otherwise wild-type adult MGs, without introducing lineage-specifying transcription factors.

**Figure 4:**
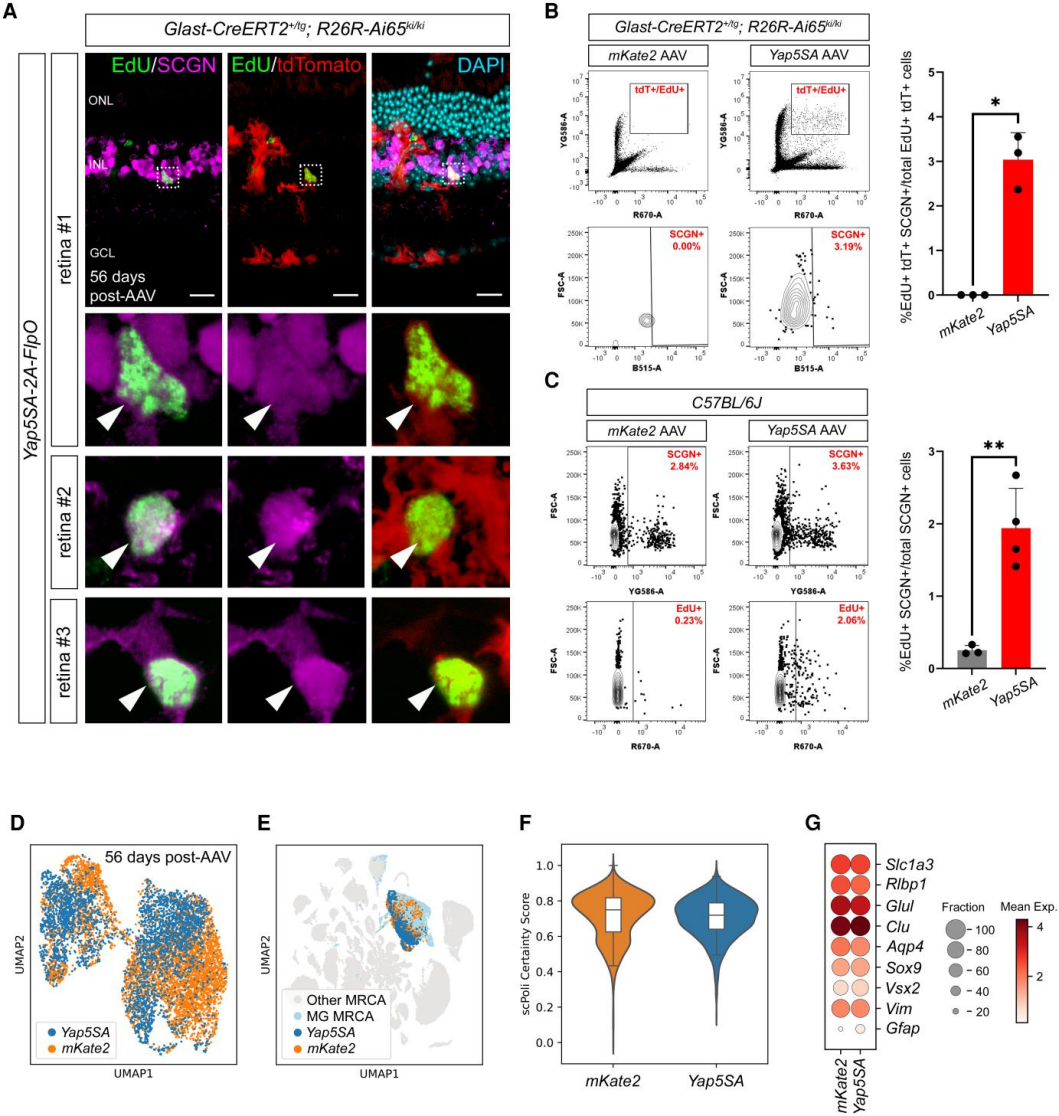
Immunofluorescent and single-cell transcriptomic profiling of MG daughter cell fate. A) IF of the bipolar cell marker SCGN showing the colocalization with a subset of EdU+/tdTomato+ cells in three independent retinas, 56 days post-*Yap5SA* AAV. B) Representative cytometry scatter plots and quantification of the % EdU+/tdTomato+/SCGN+ cells of the total EdU+/tdTomato+ population driven by *Yap5SA* AAV. C) Representative cytometry scatter plots and quantification from *C57BL/6J* mice 28 days after infection with either control or *Yap5SA* AAV, showing an increase in the % of SCGN+ cells that are also EdU+, specifically in the *Yap5SA* AAV-infected retinas. D) UMAP of integrated scRNA-seq data from FACS-sorted tdTomato+ cells from control or *Yap5SA* AAV-infected retinas. E) scPoli reference mapping of the high-quality, filtered MGs. Query cells are projected onto the MRCA latent space, overlaid onto the reference MG (MG MRCA) and background reference cells (Other MRCA). F) Distribution of the scPoli Certainty Score for the filtered query cells, demonstrating high and comparable mapping confidence to the reference MG class. White boxes indicate the interquartile range and median. G) Dot plot of average expression between conditions for canonical MG markers. Scale bars = 20 μm.

We detected no tdTomato+/EdU+ cells coexpressing HuC/D or RBPMS, arguing against amacrine or retinal ganglion cell identity (Fig. S2C–F). However, rare tdTomato+/EdU+ cells expressed the bipolar cell markers SCGN, CABP5, and PKCα (Figs. 4A and S3A–C). Flow cytometry confirmed that 3.037 ± 0.61 of tdTomato+/EdU+ cells were SCGN+ (Fig. 4B). In independent experiments using wild-type *C57BL/6J* retinas, a subset of SCGN+ cells (1.95% ± 0.58) were EdU+ exclusively in *Yap5SA*-infected retinas (Fig. 4C), further supporting bipolar cell-like differentiation. Similar enrichment was observed for CABP5 (7.99% ± 0.83) and PKCα (2.24% ± 0.81) (Fig. S3D and E). Together, these findings indicate that while YAP-induced proliferation predominantly regenerates MGs, a fraction of daughter cells acquire molecular features consistent with bipolar cell-like identity. Because this occurs without the forced expression of proneural determinants, the data suggest that YAP-driven cell-cycle reentry may transiently unlock a restricted, late progenitor-like competence state rather than induce wholesale transdifferentiation. We note, however, that this should not be interpreted as a faithful recapitulation of a normal late retinal lineage program. Although bipolar cells and MG are both late-born retinal cell types, classical lineage analyses do not support a uniquely shared sibling relationship, and rods are frequently recovered as late-born siblings (25, 26). Consistent with this distinction, we did not detect rod-like progeny in the present study.

Although immunostaining and flow cytometry supported the generation of newly derived SOX9+/LHX2+ MG and rare SCGN+, CABP5+, and PKCα+ daughter cells, marker-based analyses alone cannot establish whether these cells acquire a stable transcriptional identity. To define the molecular state of YAP5SA-induced progeny at single-cell resolution, we performed single-cell mRNA sequencing (scRNA-seq) on fluorescent-activated cell sorting (FACS)-isolated tdTomato^+^ lineage-labeled cells. Unsupervised clustering identified two principal populations (Fig. 4D), with cells from both *mKate2*- and *Yap5SA* AAV-infected retinas represented in each cluster. Quality control analysis revealed that the larger cluster, both *Yap5SA* AAV and controls, exhibited signatures consistent with dissociation-associated stress (Fig. S4). We therefore excluded this compromised population and restricted subsequent analyses to the transcriptionally robust MG cluster.

Reference mapping to the Mouse Retina Cell Atlas (MRCA) (27) using scPoli demonstrated that both *mKate2* control and *Yap5SA* lineage-labeled cells mapped to MG with high and comparable confidence (Fig. 4E and F). Canonical MG marker expression was nearly indistinguishable between conditions (Fig. 4G), and we did not detect a distinct *Scgn-*, *Cabp5-*, or *Prkca*-expressing cluster within the lineage-labeled compartment. These findings indicate that YAP5SA-induced proliferation primarily results in faithful MG self-renewal, thereby supporting *bona fide* MG regeneration. The absence of a discrete bipolar cell transcriptional program suggests that the SCGN+/CABP5+/PKCα+ cells observed by immunostaining either fall below single-cell detection thresholds or represent transient, incomplete lineage excursions rather than durable neuronal differentiation. Collectively, these data demonstrate that transient YAP activation expands the MG pool while preserving core glial identity.

In summary, transient activation of YAP in adult mammalian MGs is sufficient to induce robust, self-limited proliferation, and generate stable postmitotic progeny in the absence of injury or forced expression of neurogenic transcription factors. However, rigorous intersectional lineage tracing and single-cell transcriptomics reveal that proliferative MGs overwhelmingly undergo faithful self-renewal, with rare cells exhibiting bipolar-associated features and no evidence of sustained neuronal reprogramming. These findings resolve a central question in retinal regeneration by demonstrating that proliferative competence and neurogenic competence are mechanistically separable states in the adult mammalian retina. YAP-driven cell-cycle reentry alone is insufficient to restore embryonic-like multipotency, but it reveals a tightly regulated window of plasticity that may serve as a substrate for combinatorial reprogramming strategies. Importantly, controlled expansion of the MG population may itself confer biological advantages. An increased pool of transcriptionally authentic MGs could enhance the retina's metabolic support, structural stability, and stress-buffering capacity, potentially improving resilience to injury while preserving a homeostatic glial reservoir from which neurogenic programs might subsequently be engaged. By defining the intrinsic limits and potential benefits of MG expansion in vivo, this work establishes a mechanistic foundation for rational strategies that balance glial preservation with neuronal regeneration.

## Materials and methods

### Mouse strains and genotyping

All animal procedures were performed in accordance with protocols approved by the Institutional Animal Care and Use Committee at Baylor College of Medicine and adhered to the guidelines set forth in the ARVO Statement for the Use of Animals in Ophthalmic and Vision Research. Mice were housed in a temperature-controlled facility with a 12-h light/dark cycle and provided food and water ad libitum. *Rosa26-Ai65^+/ki^* (JAX stock #021875) (28) and *Glast-CreERT2^+/tg^* (29) mice were maintained on a *C57BL/6J* background and genotyped by PCR using established protocols. *C57BL/6J* (JAX stock #000664) mice we acquired from the Jackson lab. As no sex-related differences were observed, data from male and female animals were pooled for analysis.

### AAV construction

Using the following sequences, AAV constructs were assembled using VectorBuilder's custom cloning service and packaged into ShH10 (Addgene no. 64867) serotype vectors: *CBh* promoter (Addgene no. 105921), *Gfap* promoter (Addgene no. 50473), *EGFP* cDNA (VectorBuilder), *mKate2* cDNA (Addgene no. 105921), *FlpO* cDNA (Addgene no. 13793), and *Yap5SA* cDNA (James Martin's laboratory). Aliquots of AAV vectors were stored at −80 °C and thawed on ice immediately before use.

### Tamoxifen, EdU, and AAV injections

To activate *Glast-CreERT2*, tamoxifen (Sigma, T5648) was administered via intraperitoneal (IP) injection at a dose of 75 mg/kg body weight daily for five consecutive days. For labeling cells in S phase, 5-ethynyl-2′-deoxyuridine (EdU) (Sigma 900584) was injected IP at 50 mg/kg body weight.

For intravitreal AAV injections, mice were anesthetized with isoflurane, and one drop of 0.5% proparacaine hydrochloride was applied to the eye to be injected. The anesthetized mouse was positioned on its side on a warming pad under a dissecting microscope. A sterile 28G hypodermic needle was inserted into the postlimbal region with the bevel facing upward to avoid damaging the lens. After creating the entry site, the needle was removed, and excess vitreous fluid was gently wiped away using a sterile cotton swab. A sterile 33G blunt needle attached to a Hamilton syringe was then inserted through the puncture site, past the lens, and into the vitreous chamber to deliver 2 μL of either *mKate2* AAV (3.79 × 10^12^ genome copies/mL) or *Yap5SA* AAV (1.63 × 10^12^ genome copies/mL). The virus was slowly injected over approximately 10 seconds. Following the injection, the needle was held in place for an additional 10 s before slow withdrawal to prevent reflux. After the injection, veterinary ophthalmic antibacterial ointment (Bacitracin Zinc and Polymyxin B) was applied to the eye. Mice were monitored continuously on the warming pad until full recovery from anesthesia, indicated by the return to sternal recumbency.

### Immunofluorescence, EdU labeling, and confocal microscopy

Mice were euthanized by CO_2_ inhalation followed by thoracotomy. Dissected eyecups were fixed in 4% paraformaldehyde (PFA) for 1 h at 4 °C, followed by three 10-min phosphate-buffered saline (PBS) washes at 4 °C, then immersed in 30% sucrose at 4 °C until they sank. Tissues were subsequently embedded in optimal cutting temperature compound (OCT) and stored at −80 °C prior to sectioning. Cryosections were cut at 20 μm, postfixed in 4% PFA for 15 min, and then washed three times for 10 min each in PBS. For EdU detection, the Click-IT EdU Alexa Fluor 488 or 647 kit (Thermo Fisher) was used according to the manufacturer's instructions. For immunofluorescence, sections were incubated at room temperature for 2 h in a blocking solution containing either 2% normal goat serum or donkey serum and 3% bovine serum albumin in 0.1 M PBS (pH 7.4), followed by overnight incubation with primary antibodies in a humidified chamber at 4 °C. Slides were then rinsed three times in PBS and incubated with secondary antibodies for 2 h at room temperature in the dark. DAPI was included in the secondary antibody solution at a 1:500 dilution. Following incubation, slides were rinsed three times in PBS and mounted using Fluoromount-G (SouthernBiotech). Confocal imaging was performed using a Zeiss LSM 880 microscope with 40× objectives. To ensure consistency across samples, all images were acquired using identical settings for laser power, detector gain, scan speed, and pinhole size. Z-stack images were captured with a uniform step size and thickness.

The following primary antibodies were used: rabbit-SOX9 (1:500, Millipore, AB5535), rabbit-GFAP (1:1,000, Dako, Z0334), rabbit-KI67 (1:500, Abcam 15580), rabbit-CYCLIN D1 (1:1,000, Thermo Fisher, 9104), goat-GFP (1:1,000, NB100-1770), mouse-FLAG (1:250, Sigma, F1804), mouse-HuC/D (1:100, Invitrogen, A-21271), rabbit-RBPMS (1:500, PhosphoSolutions, 1830-RBPMS), rabbit-SCGN (1:800, Cell Signaling 15037), rabbit-PKCα (1:500, Sigma, P4334), rabbit-CABP5 (1:250, Thermo BS-12160R), rabbit-LHX2 (1:350, PA5-78287, XJ4093508C), rabbit-CASPASE 3 (1:400, BD, 559565), and rabbit-IBA1 (Invitrogen, 178847). Secondary antibodies: Alexa Fluor 488, 555, or 647-conjugated (1:400, Life Technologies).

### RNA extraction and quantitative real-time PCR

Retinas from control and experimental mice were dissected, and total RNA was extracted using the RNeasy Mini Kit (QIAGEN). Reverse transcription was carried out with the SuperScript III First-Strand Synthesis Kit (Invitrogen), using a combination of Oligo(dT)20 and random hexamer primers. Quantitative real-time PCR (qRT-PCR) was performed using the TaqMan Gene Expression Assay (Applied Biosystems) with a probe specific for *Cyclin D1* (Mm00432359). Reactions were run on a StepOnePlus Real-Time PCR System (Life Technologies) under the following cycling conditions: 50 °C for 2 min, 95 °C for 10 min, followed by 40 cycles of 95 °C for 15 s and 60 °C for 1 min.

### Western blot analyses

Dissected retinas were homogenized in ice-cold lysis buffer (Thermo Scientific 1861603) containing protease and phosphatase inhibitors and centrifuged at 4 °C (2,000 RPM, 10 min). 20 to 25 μg of clarified protein lysates were loaded onto a 4–15% Tris/Glycine precast gel (BioRad, 5671084) for electrophoresis and transferred (30 V, overnight) onto Immobilon-P PVDF membranes using the Criterion System BioRad. Membranes were washed in TBS-T pH 7.4 (20 mM Tris, 137 mM NaCl, 0.1% Tween 20) and blocked for 1 h at RT with 5% nonfat milk. At least three independent control and treated samples were probed with anti-FLAG (1:1,000, Sigma, F1804) or anti-GADPH (1:3,000, Millipore, MAB374) primary antibodies in 5% milk, overnight at 4 °C. Membranes were then washed in TBS-T, incubated with HRP-conjugated secondary antibodies (1 h, room temperature), detected with Clarity Western ECL Substrate (Bio-Rad #170-5060), and imaged with the Chemidoc Touch Imager (Bio-Rad).

### Tissue dissociation and cell sorting

Retinas were dissected in ice-cold PBS and enzymatically dissociated using 10 units of papain and 60 units of DNase (500 μL/30 μL for 3–6 retinas) for 25–30 min in a shaking water bath at 37 °C. Following digestion, tissues were gently triturated 10 times with a fire-polished pipette, and the reaction was quenched by adding EBSS containing ovomucoid and DNase. The resulting cell suspension was filtered through a 40 μm strainer and centrifuged at 900 RPM for 8–10 min.

For FACS prior to scRNA-seq, cell pellets were resuspended in a solution of DNase, ovomucoid, and Neurobasal medium (1:1:10) and kept on ice. FACS was performed using a Sony MA900 cell sorter (BD Biosciences) with an 80-micron nozzle to isolate tdTomato-positive and -negative cells. Sorted cells were collected into prechilled tubes containing 0.04% ultrapure BSA in 0.1 M PBS and immediately processed for single-cell RNA sequencing.

For flow cytometry and quantification of EdU-positive cells, cell pellets were washed with 0.1 M PBS and fixed in 2% PFA for 30 min at room temperature. After fixation, cells were permeabilized with 0.5% Triton X-100 for 30 min at room temperature, then washed with 2% BSA. EdU labeling was performed using the Click-iT Plus EdU Alexa Fluor 647 kit (ThermoFisher) according to the manufacturer's protocol. Cells were incubated in the reaction cocktail for 30 min, washed with 2% BSA, and resuspended in 0.1 M PBS. Tomato-positive and EdU-positive populations were quantified using the Symphony flow cytometer (Life Technologies).

### Single-cell mRNA sequencing

Single-cell RNA sequencing was performed using the 10× Genomics Chromium platform (10× Genomics, Pleasanton, CA). FACS-purified tdTomato+ cells from AAV-control (*n* = 16 retinas pooled) and AAV-YAP5SA-infected retinas (*n* = 21 retinas pooled) at 56 days postinjection processed immediately after sorting. Cell concentration and viability were assessed, and approximately 10,000 viable cells per condition were loaded onto the Chromium Controller for single-cell capture and barcoding using the Chromium Single Cell 3′ Reagent Kit v3.1 (10× Genomics, PN-1000269). Following reverse transcription and barcoded cDNA amplification according to the manufacturer's protocol, sequencing libraries were prepared and submitted to Admera Health (South Plainfield, NJ) for sequencing. Libraries were pooled and sequenced on an Illumina NovaSeq X Plus platform using 10B flow cells with 2 × 150 bp paired-end sequencing (NovaSeq X Plus 10B 2 × 150 configuration) to achieve a target depth of ∼50,000–100,000 reads per cell. Demultiplexing was performed by the sequencing facility, and raw sequencing data were delivered via Illumina BaseSpace with MD5 checksums and quality control summary reports.

### scRNA-seq quality control and downstream analysis

10× genomics gene expression libraries were aligned to the GRCm39 reference genome (refdata-gex-GRCm39-2024-A) using Cell Ranger v.8.0.0. The resulting count matrices were processed in Scanpy (30) to remove low-quality cells on a per-sample basis. Cells were excluded if their log-transformed total counts, log-transformed distinct genes, or percentage of counts in the top 20 highly expressed genes fell outside four median absolute deviations (MADs) from the sample median. Additionally, cells were excluded if their mitochondrial transcript fraction exceeded 15% or deviated by more than three MADs from the sample median.

To integrate the samples, we trained an scVI model (31) using default parameters on the raw counts of the 3,000 most highly variable genes, selected using the Seurat v3 dispersion method (32). A k-nearest neighbor graph (*k* = 15) was constructed from the scVI latent embedding, followed by unsupervised Leiden clustering and Uniform Manifold Approximation and Projection (UMAP) for dimensional reduction and visualization.

To optimize computational efficiency, the MRCA (27) reference dataset was first randomly down-sampled to a maximum of 8,000 cells per annotated cell class. For robust, automated cell type annotation, label transfer analysis was performed using PopV (33), with the down-sampled MRCA serving as the reference. Reference annotations at the “majorclass” level were mapped to our query cells. We retained only high-confidence MG and excluded all other predicted cell types and low-confidence assignments.

Following this filtering step, the high-quality *Yap5SA* and *mKate2* MG were projected onto the MRCA latent space. We trained a semi-supervised scPoli (34) model on the down-sampled MRCA using “majorclass” as the cell type label and sample ID as the batch covariate. The query cells were then mapped to this reference, and a UMAP was calculated using the joint latent representation. Finally, to quantify mapping confidence, the scaled scPoli uncertainty measure was inverted (1-uncertainty score) to derive an intuitive prediction certainty score for each mapped cell.

### Quantification and statistical analysis

#### Confocal image quantification

To assess colocalization of GFP+ and glutamine synthetase (GS)+ pixels as a measure of MG-specific viral transduction, line profile analysis was performed using ZEN software (Zeiss). A single representative profile line was drawn across the retinal section. Pixel intensity values for both the GFP and GS fluorescence channels were measured and plotted as a function of distance along the line, generating a dual-channel intensity profile. Spatial overlap of GFP+ and GS+ intensity peaks along the profile line was used to quantitatively assess colocalization of viral transduction with MG identity.

To quantify the number of proliferating YAP5SA+ MG-derived cells across the proliferation time course, EdU+/tdTomato+ double-positive cells were manually counted from immunostained retinal sections. For each retina, three representative retinal cross-sections were selected, and all EdU+/tdTomato+ double-positive cells visible within each section were counted. Counts from the three sections were averaged to generate a single mean value per retina, which served as the biological replicate value for statistical analysis, with two to four retinas per timepoint. This was applied across all timepoints spanning day 14 through day 56 post-AAV injection.

For all remaining quantifications, images were processed and analyzed using FIJI (ImageJ, NIH). To ensure consistency and objectivity, a manual threshold was determined by evaluating the full set of images for a given staining condition, and the same threshold was then applied uniformly to all images within that condition without further adjustment. Binary masks were generated independently for each thresholded channel. To quantify colocalization between markers (eg SOX9+ pixels overlapping with EdU+/tdTomato+ pixels), binary masks were generated for each fluorescence channel as described above. Pixel counts were determined for each individual mask. The “AND” function in FIJI was applied to compute the pixel-wise logical intersection between the SOX9+ mask and the combined EdU+/tdTomato+ mask, identifying only those pixels that were simultaneously positive in both channels. The resulting overlapping pixel count was expressed as a percentage of the total EdU+/tdTomato+ masked pixels. For each experiment, four to six independent retinas from separate mice were examined.

#### Flow cytometry gating

Flow cytometry data were analyzed using FlowJo software (BD Biosciences). Standard sequential gating was applied to all samples to eliminate cell debris and singlet discrimination to exclude doublets. Subsequent gates for populations of interest (tdTomato+, EdU+, and/or antibody+) were set based on appropriate control samples. For antibody-stained samples, a no-primary antibody control and a no-secondary antibody control were included to establish background fluorescence levels and set gates. For EdU incorporation, a no-EdU control sample was used to define the EdU-negative population and establish the EdU-positive gate. For Annexin V staining, gates were set to be more inclusive to capture cells at early stages of apoptosis; however, obvious cell debris, identified by scatter characteristics, was excluded from analysis.

#### qRT-PCR analysis

qRT-PCR data were analyzed using the Pfaffl method to account for primer-specific amplification efficiencies (35). Mean Ct values for each gene of interest were normalized to the geometric mean of the housekeeping gene, *Hprt*, to control for variation in input RNA quantity and cDNA synthesis efficiency. Each biological sample was run in technical triplicate, and the mean Ct value per sample was used for all statistical comparisons. Data were log_2_-transformed to derive ΔΔCt fold-change values.

#### Statistical analysis

All statistical analyses and graph generation were performed using GraphPad Prism (version 10 or later). Data are presented as mean ± SD with individual data points shown for all graphs. For quantification data derived from measurements taken within the same image or section (eg pixel overlap percentages), a paired Student's t test was used. For all other comparisons between *Yap5SA* AAV and *mKate2* AAV groups, an unpaired Student's t test was used. Prior to each unpaired t test, an F-test was performed to assess equality of variances between groups. If the F-test indicated significant variance differences between groups, Welch's correction was applied to the unpaired t test to account for unequal variances. If variances were not significantly different, a standard unpaired t test without Welch's correction was used. For all comparisons, a *P*-value of <0.05 was considered statistically significant. Sample sizes of four to six independent biological replicates (individual retinas from separate mice) per experimental group were used.

## Supplementary Material

pgag188_Supplementary_Data

## Data Availability

The accession number for the scRNA-seq data reported in this article is GSE322628 and is accessible through the NCBI's Gene Expression Omnibus (GEO) (36).
