# Supplementary material for: Transient YAP activation uncovers the neurogenic potential of proliferative mammalian Müller glia
Source: PNAS Nexus. 2026 May 28;5(6):pgag188. doi: 10.1093/pnasnexus/pgag188 (PMC13273572; doi:10.1093/pnasnexus/pgag188)
Supplement: pgag188_Supplementary_Data [file pgag188_Supplementary_Data.pdf]

SUPP. FIGURE 1

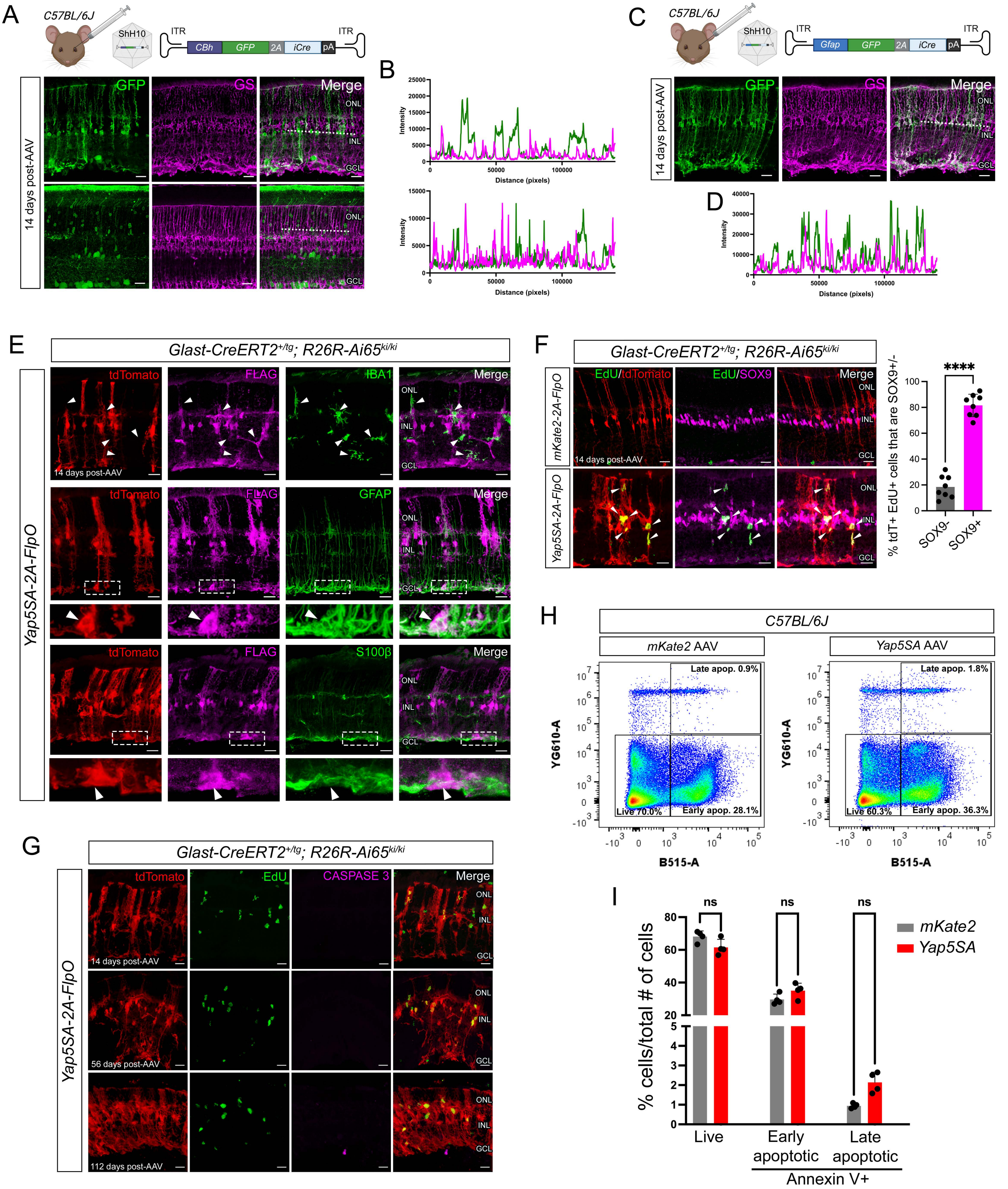

Supplemental Figure 1. (A) Top: Schematic of the constitutive *CBh* promoter-driven GFP packaged in the ShH10 capsid and delivered intravitreally. Bottom: Confocal microscopy image showing significant off-target GFP expression outside of the MGs 14 days post-AAV infection. **(B)** Line profile analysis of GFP and GS fluorescence intensity across the retinal section showing non-overlapping peaks. **(C)** Top: Schematic of the constitutive *Gfap* promoter-driven GFP packaged in the ShH10 capsid and delivered intravitreally. Bottom: Confocal microscopy image showing greatly improved on-target GFP expression within the MG population 14 days post-AAV infection. **(B)** Line profile analysis of GFP and GS fluorescence intensity across the retinal section, showing overlapping peaks. **(E)** IF from $CreERT2^{+/tg}$; $R26R\text{-}Ai65^{ki/ki}$ retinas 14 days post-*Yap5SA* AAV injection showing no co-localization of tdTomato+/FLAG+ cells with the microglial marker IBA1 and astrocyte markers GFAP and S100β. **(F)** IF at 14 days post-AAV injection and pixel quantification of EdU, tdTomato, and SOX9 showing EdU+ tdTomato+ cells co-localize with SOX9+ MG. **(G)** IF for activated CASPASE 3 showing no co-localization with EdU+/tdTomato+ cells 14, 56, and 112 days after infection with *Yap5SA* AAV. **(H)** Cytometry from *mKate2* AAV and *Yap5SA* AAV-infected *C57BL/6J* retinas stained with Annexin V and propidium iodide (PI). **(I)** Quantification indicating no significant increase in apoptosis due to *Yap5SA* AAV. All quantified data are shown as mean ± SD and $n \geq 3$ biologically independent samples per group. Significant differences between groups were determined using a paired t-test (F) or an unpaired t-test with Welch correction (I). A p-value of < 0.05 was considered significant. Scale bars = 20 µm. $^{*}p < 0.05$, $^{**}p < 0.01$, $^{***}p < 0.001$, $^{****}p < 0.0001$.Created in BioRender. Poché, R. (2026) https://BioRender.com/44pbtxf

SUPP. FIGURE 2

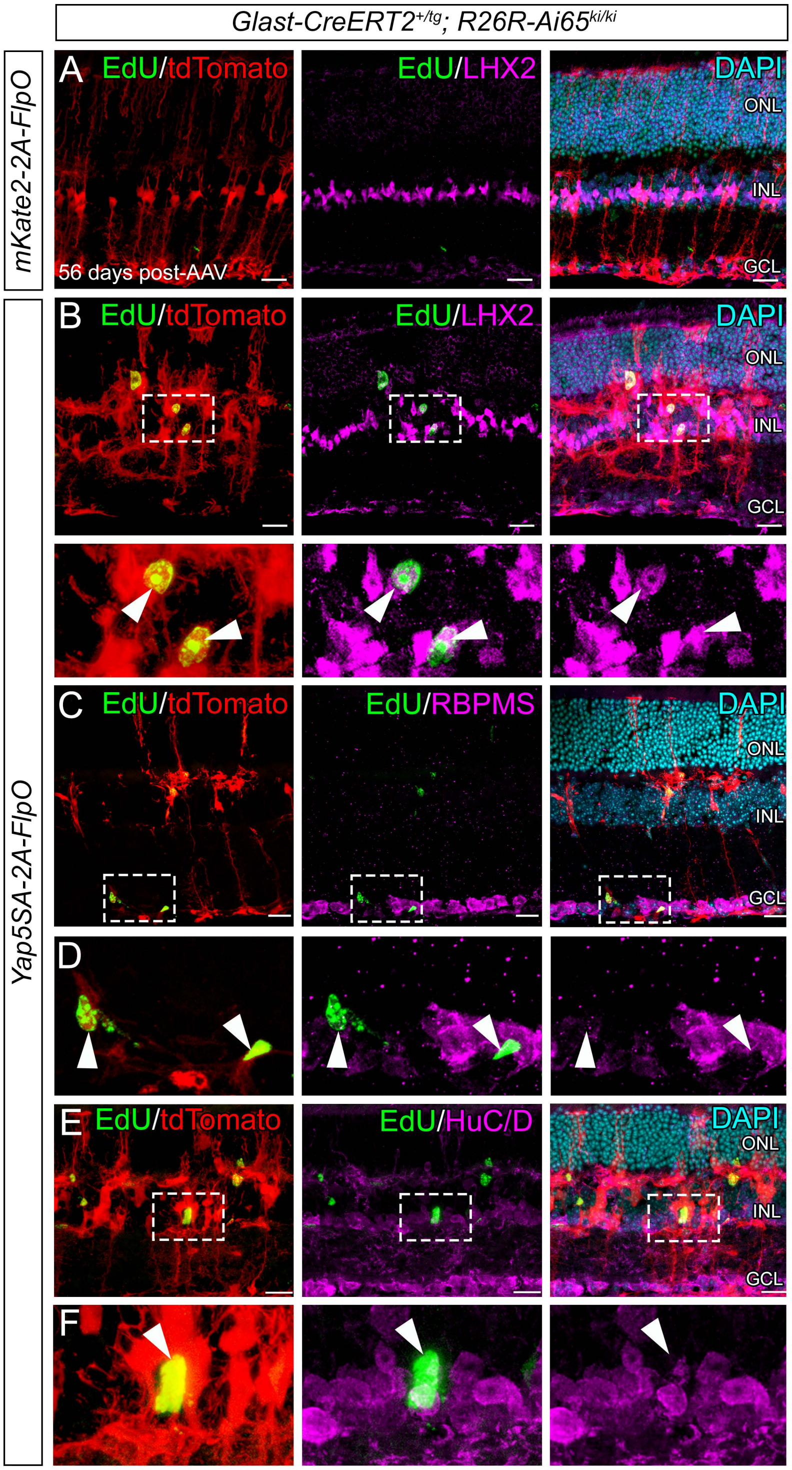

Supplemental Figure 2. (A-B) IF showing that *Yap5SA* AAV-derived EdU+/tdTomato+ cells express the MG marker LHX2, but not ganglion and amacrine markers **(C-D)** RBPMS or **(E-F)** HuC/D 56 days post-AAV infection (boxed regions and arrowheads). Scale bars = 20 μm.

SUPP. FIGURE 3

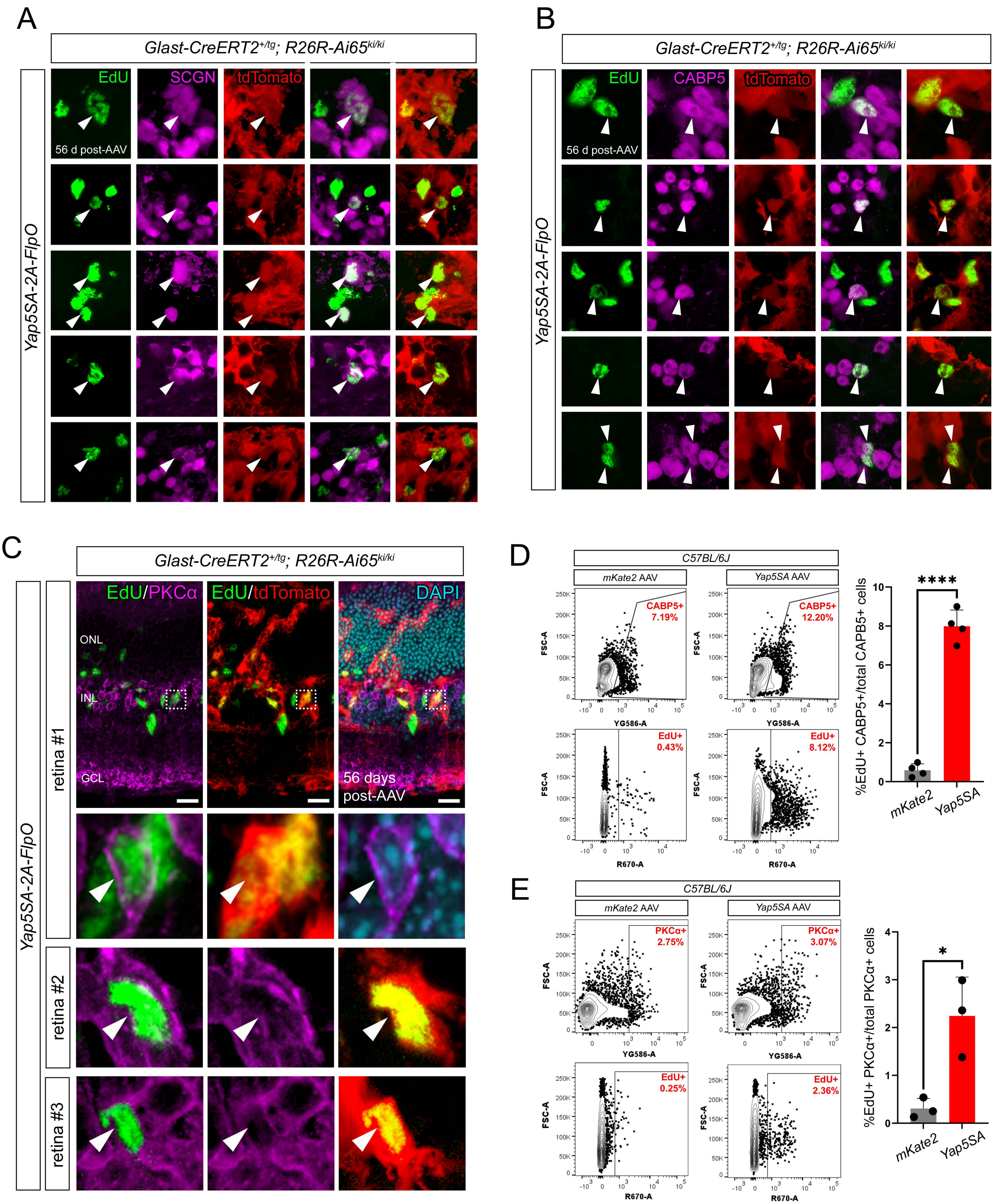

Supplemental Figure 3. (A-C) IF showing high magnification examples of *Yap5SA* AAV-derived EdU+/tdTomato+ cells that express the bipolar cell markers SCGN **(A)**, CABP5 **(B)**, and PKCα **(C)** 56 days post-*Yap5SA* AAV infection (arrowheads). **(D-E)** Cytometry from *C57BL/6J* mice 28 days after infection with either *mKate2* or *Yap5SA* AAV, showing an increase in the % of CABP5 + cells **(D)** and PKCα+ cells **(E)** that are also EdU+, specifically in the *Yap5SA* AAV-infected retinas. All quantified data are shown as mean ± SD and n ≥ 3 biologically independent samples per group. Significant differences between groups were determined using an unpaired t-test. A p-value of < 0.05 was considered significant. Scale bars = 20 µm. *p < 0.05, **p < 0.01, ***p < 0.001, ****p < 0.0001.

SUPP. FIGURE 4

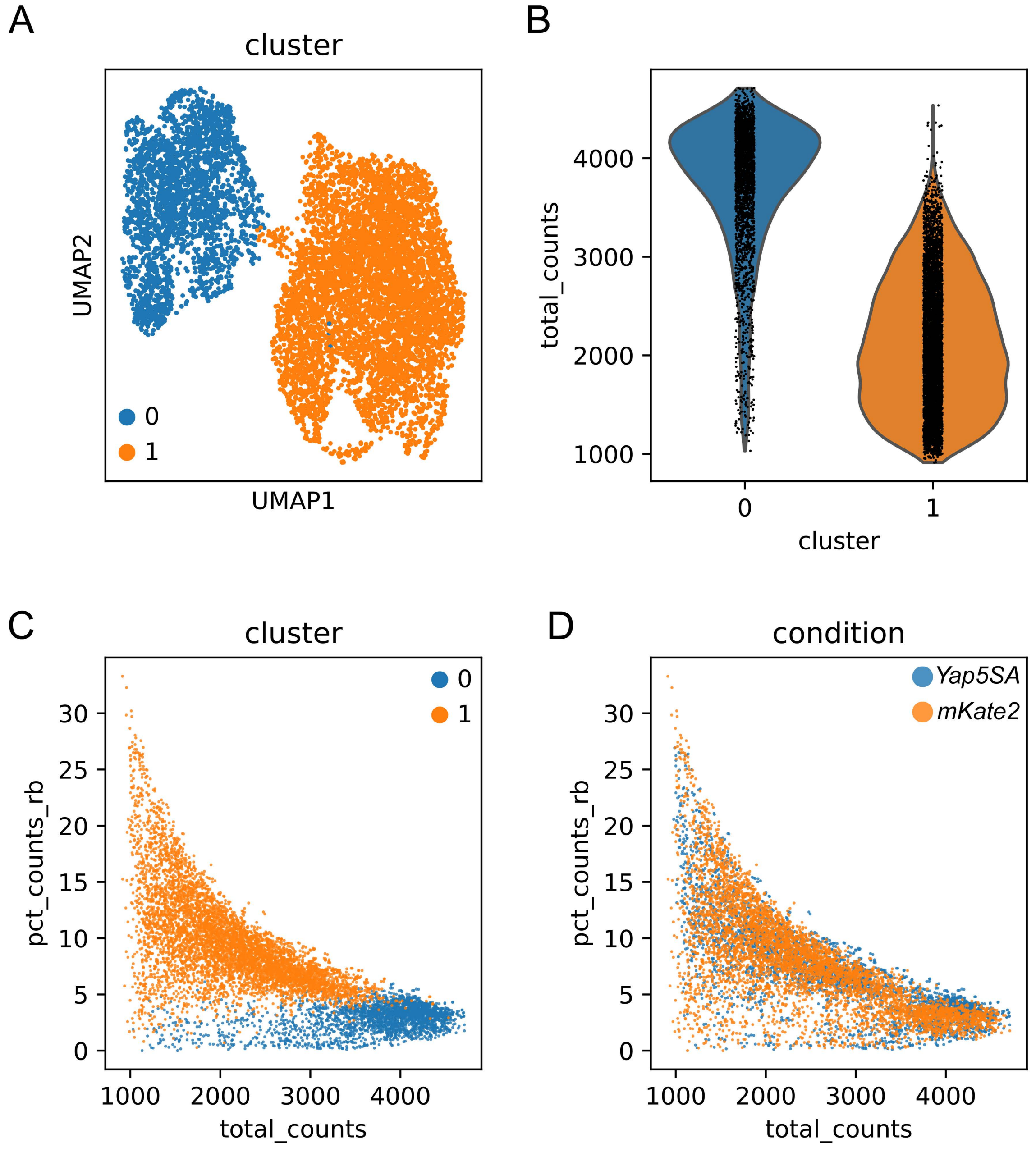

Supplemental Figure 4. (A) UMAP of integrated scRNA-seq data from FACS-sorted tdTomato+ MGs, displaying two distinct groups colored by unsupervised cluster assignments. **(B)** Violin plot showing the distribution of total UMI counts. Cluster 1 exhibits a noticeably lower distribution of counts. **(C)** Scatter plot comparing UMI counts to the percentage of ribosomal gene counts, colored by cluster. Cluster 1 represents a stressed population characterized by lower transcriptomic complexity and elevated ribosomal gene expression. **(D)** Same as C, colored by experimental condition. Cells infected with both *Yap5SA* AAV and *mKate2* AAV are equally distributed across the stressed population.
